# Prognostic role of proliferating CD8^+^ cytotoxic Tcells in human cancers

**DOI:** 10.1007/s13402-021-00601-4

**Published:** 2021-04-17

**Authors:** Niclas C. Blessin, Wenchao Li, Tim Mandelkow, Hannah L. Jansen, Cheng Yang, Jonas B. Raedler, Ronald Simon, Franziska Büscheck, David Dum, Andreas M. Luebke, Andrea Hinsch, Katharina Möller, Anne Menz, Christian Bernreuther, Patrick Lebok, Till Clauditz, Guido Sauter, Andreas Marx, Ria Uhlig, Waldemar Wilczak, Sarah Minner, Till Krech, Christoph Fraune, Doris Höflmayer, Eike Burandt, Stefan Steurer

**Affiliations:** 1 Institute of Pathology, University Medical Centre Hamburg-Eppendorf, Martinistraße 52, D-20246 Hamburg, Germany; 2 College of Arts and Sciences, Boston University, Boston, MA USA; 3 Institute of Pathology, Medical Centre Fürth, D-90766 Fürth, Germany

**Keywords:** CD8^+^ cytotoxic Tcells, Tumor microenvironment, Colorectal cancer, Renal cell cancer, Breast cancer, Pancreatic cancer, Gastric cancer, Ovarian cancer

## Abstract

**Purpose:**

Expansion of CD8^+^ cytotoxic Tlymphocytes is a prerequisite for anti-cancer immune activity and has gained interest in the era of immune checkpoint therapy.

**Methods:**

To understand the CD8^+^ T cell dynamics in the tumor microenvironment, we used multiplex fluorescence immunohistochemistry to quantitate CD8^+^ proliferation (Ki67 co-expression) in tissue microarrays from 1107 colorectal, 642 renal cell, 1066 breast, 375 ovarian, 451 pancreatic and 347 gastric cancer samples.

**Results:**

The density and the percentage of proliferating (Ki67^+^) CD8^+^ T cells were both highly variable between tumor types as well as between patients with the same tumor type. Elevated density and percentage of proliferating CD8^+^ cytotoxic T cells were significantly associated with favorable tumor parameters such as low tumor stage, negative nodal stage (*p* ≤ 0.0041 each), prolonged overall survival (*p* ≤ 0.0028 each) and an inflamed immune phenotype (*p* = 0.0025) in colorectal cancer and, in contrast, linked to high tumor stage, advanced ISUP/Fuhrman/Thoenes grading (each *p* ≤ 0.003), shorter overall survival (*p* ≤ 0.0330 each) and an immune inflamed phenotype (*p* = 0.0094) in renal cell cancer. In breast, ovarian, pancreatic and gastric cancer the role of (Ki67^+^)CD8^+^ Tcells was not linked to clinicopathological data.

**Conclusion:**

Our data demonstrate a tumor type dependent prognostic impact of proliferating (Ki67^+^)CD8^+^ Tcells and an inverse impact in colorectal and renal cell cancer.

**Supplementary Information:**

The online version contains supplementary material available at 10.1007/s13402-021-00601-4.

## Introduction

Tumor infiltrating lymphocytes (TILs) occur at variable frequencies in human cancers [1]. For many tumor types, a high number of TILs has been shown to be linked to a favorable prognosis [2–7] and a high likelihood of response to immune checkpoint inhibitors [6, 8–10]. It is generally assumed that CD8^+^ cytotoxic T-lymphocytes represent the most important TIL subset, as these cells can directly kill cancer cells [11–13]. High CD8^+^ T cell densities were found to be associated with a favorable prognosis in various tumor entities, including melanoma, colorectal cancer, breast cancer, hepatocellular cancer and non-small cell lung cancer [2, 5, 12–15]. However, the link between elevated CD8^+^ T cell density and favorable outcome may not hold true for all cancer types. For example, data from two studies on 221 and 135 cancers have suggested a poor patient outcome in case of renal cell cancers (RCCs) with elevated densities of CD8^+^ cells [16, 17]. It is assumed, that besides the mere presence of CD8^+^ cells in the cancer microenvironment, specific subsets of these cells may be functionally relevant. To obtain clinically relevant information, it may thus be necessary to measure distinct CD8^+^ T cell subsets, such as active and expanding CD8^+^ cells [14, 16, 18].

The Ki67 protein can be utilized for this purpose. Ki67 is expressed in all proliferating cells in G1, S, G2 and M phases of the cell cycle [19, 20]. Thus, the Ki67^+^ expanding CD8^+^ T cell subset has been proposed to represent an activated CD8^+^ T cell subset [21]. The role of CD8^+^Ki67^+^ T lymphocytes may also vary between tumor entities. Studies in rectal, renal cell and cervical cancer, as well as in malignant melanoma have proposed that CD8^+^Ki67^+^ Tlymphocytes may be prognostically relevant in rectal and RCC but not in cervical cancer and malignant melanoma [14, 16, 22, 23]. However, as different approaches were used for cell quantification in these studies, it cannot be excluded that perceived differences between tumor entities may be attributable to methodological differences.

This study was designed to assess the potential clinical significance of CD8^+^Ki67^+^ T lymphocytes in multiple relevant cancer types by using one standardized approach. For this purpose, cohorts of colorectal, renal cell, breast, gastric, ovarian and pancreatic cancer samples comprising more than 3500 tumors were analyzed in a tissue microarray (TMA) format using a standardized quantification algorithm for multiplex-fluorescence immunohistochemistry staining.

## Materials and methods

### Tissues and tissue microarrays

Formalin-fixed paraffin-embedded tissue samples of 6441 patients were selected from the archives of the Institute of Pathology of the University Medical Center Hamburg-Eppendorf, Germany. The construction of tissue microarrays (TMAs) was previously described [24]. Six different sets of TMAs were analyzed containing one 0.6 mm sample each from 1475 colorectal cancers, 1566 breast cancers, 1809 RCCs, 607 ovarian cancers, 599 pancreatic cancers and 384 gastric cancers. Data on clinical follow-up were available for 3330 patients and on histological phenotype for 5976 patients. The exact composition of these TMAs is presented in supplementary Tables 1–6. The use of archived diagnostic left-over tissues for the manufacturing of TMAs and their analysis for research purposes has been approved by local laws (HmbKHG, §12,1) and by the local ethics committee (Ethics commission Hamburg, WF-049/09). All work was performed in compliance with the Helsinki Declaration.

### Immunohistochemistry

Freshly cut 4 μm tissue sections were used for multiplex fluorescence immunohistochemistry (IHC) analysis. Antibodies directed against Ki67 (Cat. #DIA-670-P1, Dianova, mouse monoclonal antibody, Clone Ki-67P, dilution 1:200) and CD8 (Cat. #DIA-TC8, Oncodianova, mouse monoclonal antibody, Clone TC8, dilution 1:200) were used to identify proliferating cytotoxic T lymphocytes. The OPAL dye kit (Cat. # NEL811001KT, AKOYA Biosciences, Menlo Park, CA, USA) was used for antibody detection. The experimental procedure was performed mainly according to the manufacturer’s instructions (Akoya). Slides were initially boiled in an autoclave (30 min at 100–120 °C in pH 9 buffer) for antigen retrieval. Antibodies to detect Ki67 and CD8 were combined with DAPI staining in each experiment. One cycle of antibody staining included peroxidase blocking, application of the first primary (Ki67) antibody, detection with a secondary HRP-conjugated antibody, fluorescence dye detection (Opal 570) and removal of the bound antibodies by microwave treatment (4 min at 100 °C and 5 min at a mean temperature of 93 °C). This cycle was repeated for the second primary (CD8) antibody and the second fluorescence dye (Opal 690). Slides were subsequently counterstained with diamidino-2-phenylindole (DAPI) and mounted in antifade solution.

### Quantification of proliferating CD8^+^ lymphocytes

**Fig. 1:**
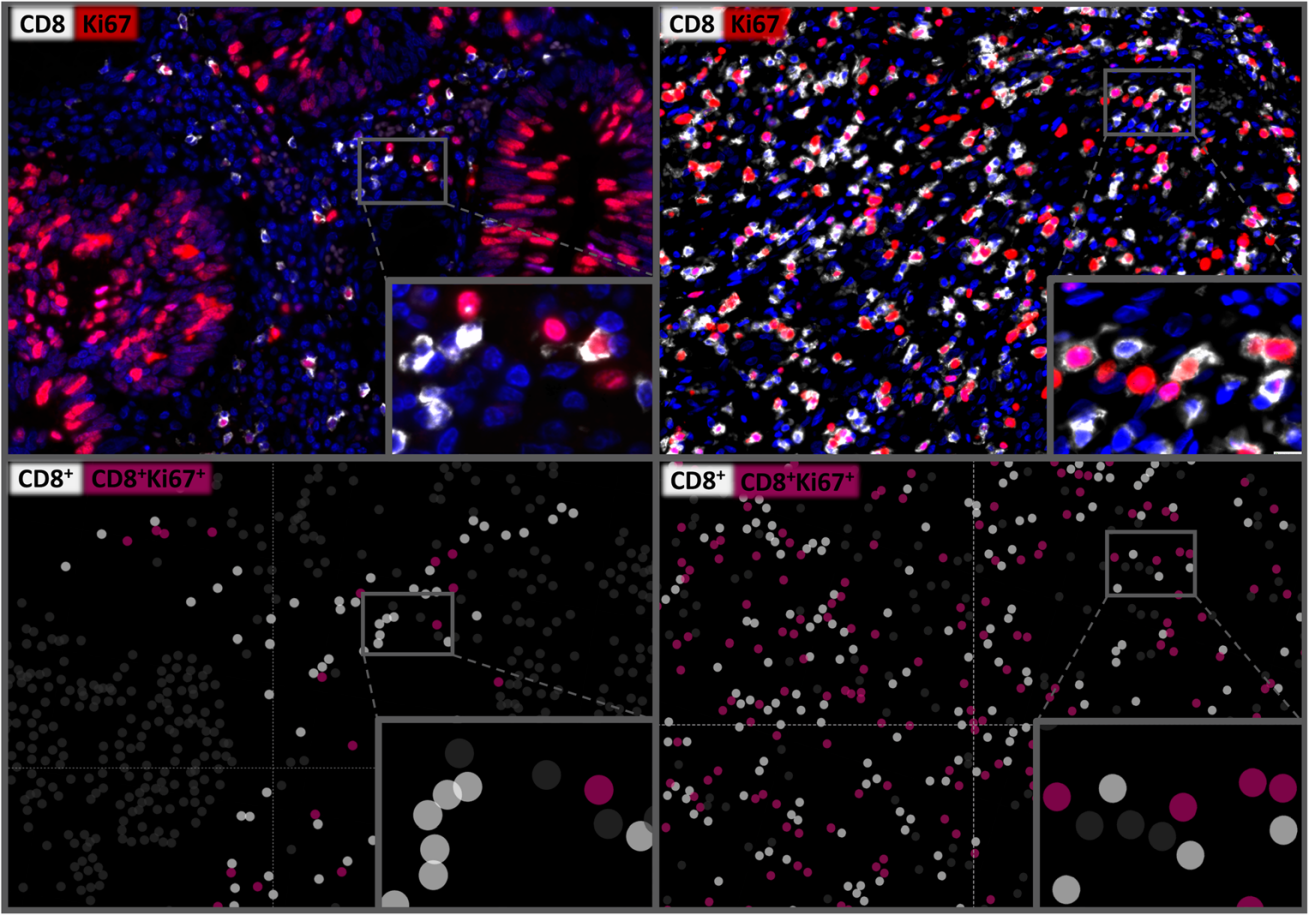
Multiplex fluorescence IHC images (top) showing CD8^+^ cytotoxic Tcells (white) and Ki67^+^ proliferating cells (red) with a low (left) and a high (right) CD8^+^Ki67^+^ proliferation rate. The visualization (bottom) shows the results of automated cell segmentation based on digital image analysis for assessing the proliferating CD8^+^Ki67^+^ cytotoxic T cell subset (purple). 400x magnifications are shown in the insets

Digital images of fluorescence stained slides were acquired using a Leica Aperio VERSA 8 automated epifluorescence microscope. Image analysis was performed using HALO™ software package (Indica Labs, USA) and included segmentation of individual cells to detect expression in Ki67^+^ and CD8^+^ cells. A visually adjusted intensity threshold was set for each marker. Cells showing staining intensities above this threshold were considered “positive”. Representative analysis results are shown in Fig. 1 and in Supplementary Fig. 1. For TMA analysis, spots were automatically identified and segmented using the HALO™ (Indica Labs, US) TMA module. Two main parameters were measured for every individual tissue compartment: (1) The density of CD8^+^ or CD8^+^Ki67^+^ stained cells per square millimeter was calculated by dividing the number of CD8^+^ or CD8^+^Ki67^+^ cells by the measured area of each tissue spot. (2) The percentage of proliferating CD8^+^ cells was calculated by dividing the number of Ki67^+^CD8^+^ double positive cells by the total number of CD8^+^ cells. A representative example of the image analysis procedure is given in Supplementary Fig. 1.

### Statistical analysis

JMP Pro 14 software package (SAS Institute Inc., NC, USA) and R version 3.6.1 (The R foundation) [25, 26] were used. All *p*-values were two-sided, and *p*-values < 0.05 were considered as significant. To study the relationship between cell densities or fractions of proliferating cells and clinicopathological parameters, Pearson correlation coefficient, contingency tables and Chi-square test (likelihood) were used. Analysis on overall survival, recurrence-free and disease-specific survival was performed using the Kaplan-Meier method and was compared via log-rank test. Cox regression and multivariate analyses were used to assess independence of the CD8^+^ and CD8^+^Ki67^+^ cell densities.

## Results

The density of CD8^+^ T cells (CD8^+^ density), the density of CD8^+^Ki67^+^ T cells (CD8^+^Ki67^+^ density) T cells and the Ki67 labeling index of CD8^+^ cytotoxic T cells/percentage of Ki67^+^ proliferating CD8^+^ cytotoxic T lymphocyte (CD8^+^Ki67^+^ proliferation rate) were successfully analyzed in a total of 1107 of 1475 (75%) colorectal, 1066 of 1566 (68%) breast, 642 of 1809 (36%) renal cell, 375 of 608 (62%) ovarian, 451 of 599 (75%) pancreatic and 347 of 384 (90%) gastric cancer samples. The remaining cases were excluded due to lack of tissue spots on the TMA sections.

### CD8^+^ T cell density

**Fig. 2:**
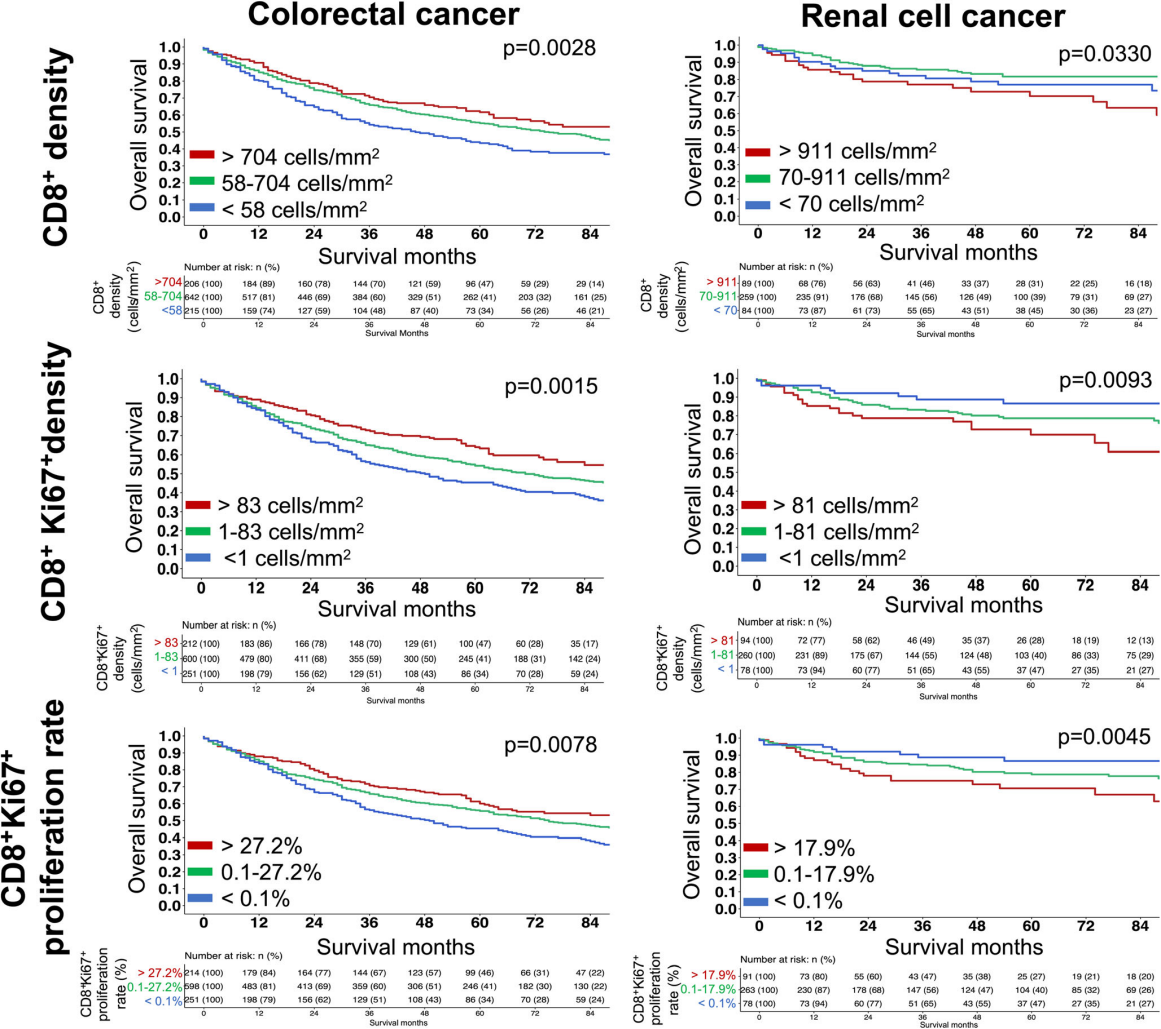
Association between CD8^+^ cell density (top), CD8^+^Ki67^+^ cell density (middle), CD8^+^Ki67^+^ proliferation rate (bottom) and overall survival in colorectal cancer (left) and renal cell cancer (right)

The density of CD8^+^ cytotoxic T lymphocytes/mm^2^ varied between different tumor entities (Fig. 1) as well as between individual patients within the same tumor entity. For example, the mean CD8^+^ T cell density ranged from 256 ± 462 cells/mm^2^ in ovarian and 288 ± 482 cells/mm^2^ in pancreatic cancer to 520 ± 887 cells/mm^2^ in colorectal and 649 ± 1077 cells/mm^2^ in renal cell cancer. The clinical significance of these findings also varied between tumor entities. Elevated CD8^+^ densities were associated with favorable tumor parameters in colorectal and gastric cancers. These included low tumor stage, negative nodal stage (*p* ≤ 0.0014 each, Table 1) and longer survival (*p* = 0.0028, Fig. 2) in colorectal cancer and lack of distant metastasis (*p* = 0.0212, Table 2) in gastric cancer. In contrast, a high CD8^+^ cell density was linked to advanced ISUP/ Fuhrman/ Thoenes grading (each *p* < 0.0001, Table 1) and poor outcome (overall survival *p* = 0.033, Fig. 2; progression free survival *p* = 0.00047, Supplementary Fig. 2) in renal cell cancer. No associations with clinicopathological parameters were found in breast, ovarian and pancreatic cancer (Table 2).

**Table 1:** Association between CD8^+^ cell density, CD8^+^Ki67+ cell density, CD8^+^Ki67^+^ proliferation rate and clinicopathological phenotype of 1107 colorectal and 642 renal cell cancer samples

Entity	Clinical parameter	n	Density of CD8^+^ cells [cells/mm^2^]	*p* value	Density of CD8^+^ Ki67^+^ cells [cells/mm^2^]	*p* value	Percentage of proliferating CD8^+^ cells [%]	*p* value
Colorectal cancer	pT1	49	1058 (±1319)	< 0.0001	271 (±625)	0.0002	19.2 (±19.2)	0.0007
	pT2	157	565 (±853)		117 (±288)		18.8 (±16.5)	
	pT3	684	481 (±845)		88 (±282)		14.2 (±16.6)	
	pT4	153	417 (±744)		66 (±153)		12.2 (±15.4)	
	pN-	558	588 (±962)	0.0014	121 (±329)	0.0041	17.1 (±17.8)	< 0.0001
	pN+	467	415 (±716)		68 (±238)		12.4 (±14.9)	
	G1	22	983 (±1321)	0.0104	308 (±814)	0.0032	19.0 (±21.5)	0.0512
	G2	896	517 (±884)		94 (±284)		14.4 (±16.4)	
	G3	124	385 (±595)		86 (±186)		17.8 (±16.7)	
	**All (±SD)**	**1107**	**520 (±887)**		**95 (±291)**		**14.6 (±16.5)**	
Renal cell cancer	pT1	332	516 (±818)	0.0030	52 (±128)	0.0003	8.4 (±10.4)	< 0.0001
	pT2	83	811 (±1295)		141 (±432)		10.8 (±9.4)	
	pT3	180	865 (±1347)		182 (±525)		14.0 (±14.2)	
	pT4	9	600 (±1236)		282 (±675)		17.7 (±23.2)	
	pN-	79	519 (±765)	0.1644	70 (±131)	0.0225	12.0 (±11.7)	0.0095
	pN+	28	854 (±1701)		252 (±669)		19.3 (±14.8)	
	ISUP 1	132	395 (±646)	< 0.0001	29 (±61)	< 0.0001	7.5 (±9.1)	< 0.0001
	ISUP 2	202	536 (±1000)		76 (±274)		9.4 (±10.5)	
	ISUP 3	190	898 (±1241)		133 (±319)		11.5 (±11.8)	
	ISUP 4	56	1172 (±1481)		349 (±818)		19.0 (±17.8)	
	Fuhrman 1	24	325 (±335)	< 0.0001	28 (±39)	< 0.0001	7.0 (±7.4)	< 0.0001
	Fuhrman 2	300	506 (±922)		63 (±230)		8.8 (±10.2)	
	Fuhrman 3	202	851 (±1140)		117 (±255)		11.1 (±11.6)	
	Fuhrman 4	58	1195 (±1657)		367 (±864)		19.5 (±17.3)	
	Thoenes 1	175	432 (±714)	< 0.0001	46 (±158)	< 0.0001	8.5 (±9.7)	< 0.0001
	Thoenes 2	317	720 (±1115)		92 (±259)		9.5 (±10.7)	
	Thoenes 3	92	1051 (±1490)		296 (±715)		18.3 (±16.0)	
	**All (±SD)**	**642**	**649 (±1077)**		**101 (±346)**		**10.5 (±12.0)**	

*pT* pathological tumor stage, *pN* pathological nodal stage, *G* histologic grade

**Table 2:** Association between CD8+ cell density, CD8+Ki67+ cell density, CD8^+^Ki67^+^ proliferation rate and clinicopathological phenotype of 1066 breast, 347 gastric, 375 ovarian and 451 pancreatic cancer samples

Entity	Clinical parameter	n	Density of CD8^+^ cells [cells/mm^2^]	*p* value	Density of CD8^+^ Ki67^+^ cells [cells/mm^2^]	*p* value	Percentage of proliferating CD8+ cells [%]	*p* value
Breast cancer	pT1	538	459 (±985)	0.5892	49 (±161)	0.1400	8.4 (±12.6)	0.0097
	pT2	397	475 (±836)		62 (±174)		10.7 (±13.4)	
	pT3	37	556 (±949)		111 (±341)		8.8 (±11.1)	
	pT4	50	307 (±570)		38 (±94)		13.3 (±16.3)	
	pN-	442	432 (±894)	0.8640	43 (±126)	0.8931	8.7 (±13.5)	0.6003
	pN+	320	444 (±875)		44 (±140)		8.3 (±11.2)	
	G1	163	210 (±396)	< 0.0001	16 (±32)	< 0.0001	8.9 (±14.4)	< 0.0001
	G2	553	333 (±686)		25 (±61)		7.9 (±11.4)	
	G3	346	783 (±1223)		125 (±281)		13.1 (±14.6)	
	PD-L1 negative	711	387 (±730)	< 0.0001	36 (±106)	< 0.0001	9.0 (±12.5)	< 0.0001
	PD-L1 weak	51	1540 (±1912)		274 (±370)		20.3 (±18.3)	
	PD-L1 moderate	7	3111 (±1349)		976 (±645)		30.2 (±12.3)	
	PD-L1 strong	2	4041 (±1208)		1263 (±823)		29.5 (±11.5)	
	**All (±SD)**	**1066**	**464 (±901)**		**56 (±173)**		**9.6 (±13.2)**	
Gastric cancer	pT1	27	442 (±629)	0.8397	111 (±133)	0.7345	29.7 (±18.3)	0.1175
	pT2	42	352 (±379)		84 (±118)		25.1 (±23.6)	
	pT3	141	394 (±553)		139 (±305)		25.4 (±24.4)	
	pT4	125	447 (±835)		138 (±367)		20.1 (±21.4)	
	pN-	86	472 (±631)	0.3374	156 (±283)	0.3593	27.1 (±23.3)	0.1303
	pN+	247	393 (±673)		121 (±312)		22.7 (±22.7)	
	M0*	275	458 (±704)	0.0212	146 (±318)	0.0970	24.4 (±22.5)	0.3251
	M1*	47	214 (±369)		65 (±243)		20.8 (±24.4)	
	HER2-	280	405 (±608)	0.1179	122 (±265)	0.1928	23.6 (±22.7)	0.8943
	HER2+	23	206 (±176)		50 (±56)		22.9 (±21.9)	
	**All (±SD)**	**347**	**412 (±654)**		**128 (±300)**		**23.5 (±22.6)**	
Ovarian cancer	pT1	63	213 (±405)	0.6907	60 (±153)	0.4623	20.8 (±19.5)	0.0327
	pT2	45	286 (±527)		60 (±86)		26.1 (±20.5)	
	pT3	209	253 (±439)		86 (±201)		28.9 (±22.2)	
	pN-	96	282 (±552)	0.8713	94 (±263)	0.8578	26.3 (±21.6)	0.4271
	pN+	136	293 (±487)		99 (±207)		28.6 (±21.2)	
	**All (±SD)**	**375**	**256 (±462)**		**77 (±190)**		**25.8 (±21.6)**	
Pancreatic cancer	pT1	15	329 (±474)	0.3195	20 (±50)	0.2652	4.5 (±5.1)	0.0379
	pT2	70	359 (±570)		40 (±100)		10.2 (±10.8)	
	pT3	325	275 (±476)		25 (±45)		12.2 (±13.8)	
	pT4	30	174 (±213)		26 (±56)		15.9 (±16.7)	
	pN-	92	316 (±427)	0.4421	35 (±91)	0.1295	10.7 (±12.8)	0.3815
	pN+	343	273 (±493)		25 (±45)		12.0 (±13.5)	
	G1	16	177 (±173)	0.3320	11 (±16)	0.0972	7.8 (±13.7)	0.0086
	G2	314	306 (±525)		26 (±45)		10.9 (±12.4)	
	G3	97	240 (±376)		38 (±93)		15.4 (±16.1)	
	**All (±SD)**	**451**	**288 (±482)**		**29 (±68)**		**12.0 (±13.8)**	

*pT* pathological tumor stage, *pN* pathological nodal stage, *G* histologic grade, M* clinical metastasis stage

### Density and percentage of Ki67^+^ proliferating CD8^+^ Tcells

A similar variability, as seen for the CD8^+^ cell density, was also found for the CD8^+^Ki67^+^ proliferation rate (Fig. 1). The mean density of CD8^+^Ki67^+^ cells ranged from 29 ± 68 cells/mm^2^ in pancreatic cancer to 128 ± 300 cells/mm^2^ in gastric cancer, while the mean CD8^+^Ki67^+^ proliferation rate ranged from 9.6 ± 13.2% in breast cancer to 25.8 ± 21.6% in ovarian cancer. An increased density and proliferation rate of CD8^+^Ki67^+^ T cells was significantly associated with low tumor stage, negative nodal stage (*p* ≤ 0.0041 each, Table 1) and longer overall survival (*p* ≤ 0.0078 each, Fig. 2) in colorectal cancer. In contrast, an elevated density and proliferation rate of CD8^+^Ki67^+^ T cells was significantly associated with high tumor stage, positive nodal stage, advanced ISUP, Fuhrman, Thoenes grading (each *p* ≤ 0.0225, Table 1) as well as shorter overall survival (*p* ≤ 0.0093 each, Fig. 2) in renal cell cancer. No associations with clinicopathological parameters were found in gastric, breast, ovarian and pancreatic cancer (Table 2).

### Combined analysis

Combined analysis of the CD8^+^ density and the CD8^+^Ki67^+^ density revealed a significant correlation of both parameters in colorectal, renal cell, breast, ovarian, pancreatic and gastric cancer (r: 0.54 to 0.81, *p* < 0.0001 each). In addition, the CD8^+^ density and the CD8^+^Ki67^+^ proliferation rate showed a significant correlation in colorectal, breast, renal cell, ovarian and gastric cancer (r: 0.01 to 0.26 *p* ≤ 0.029 each). In colorectal cancer, the multivariate analysis revealed that tumor stage (*p* < 0.0001), nodal stage (*p* < 0.0001) as well as the density of CD8^+^ (*p* = 0.0132) and the CD8^+^Ki67^+^ T lymphocyte density (*p* = 0.0098) were independent risk factors, while the percentage of proliferating CD8^+^ cells was not an independent predictor (*p* = 0.23). Additional Cox regression analysis showed that the combination of nodal stage and CD8^+^ cell density (AUC: 0.731 [0.696,0.766]) was significantly superior compared to pN alone (AUC: 0.712, [0.681,0.744], *p* = 0.029). Moreover, the combination of nodal stage and CD8^+^Ki67^+^ cell density (AUC: 0.732 [0.698,0.767]) showed an improved predictive value compared to nodal stage alone (0.712 [0.681, 0.744], *p* = 0.019).

### Cluster analysis of the CD8^+^ density and proliferation rate

**Fig. 3:**
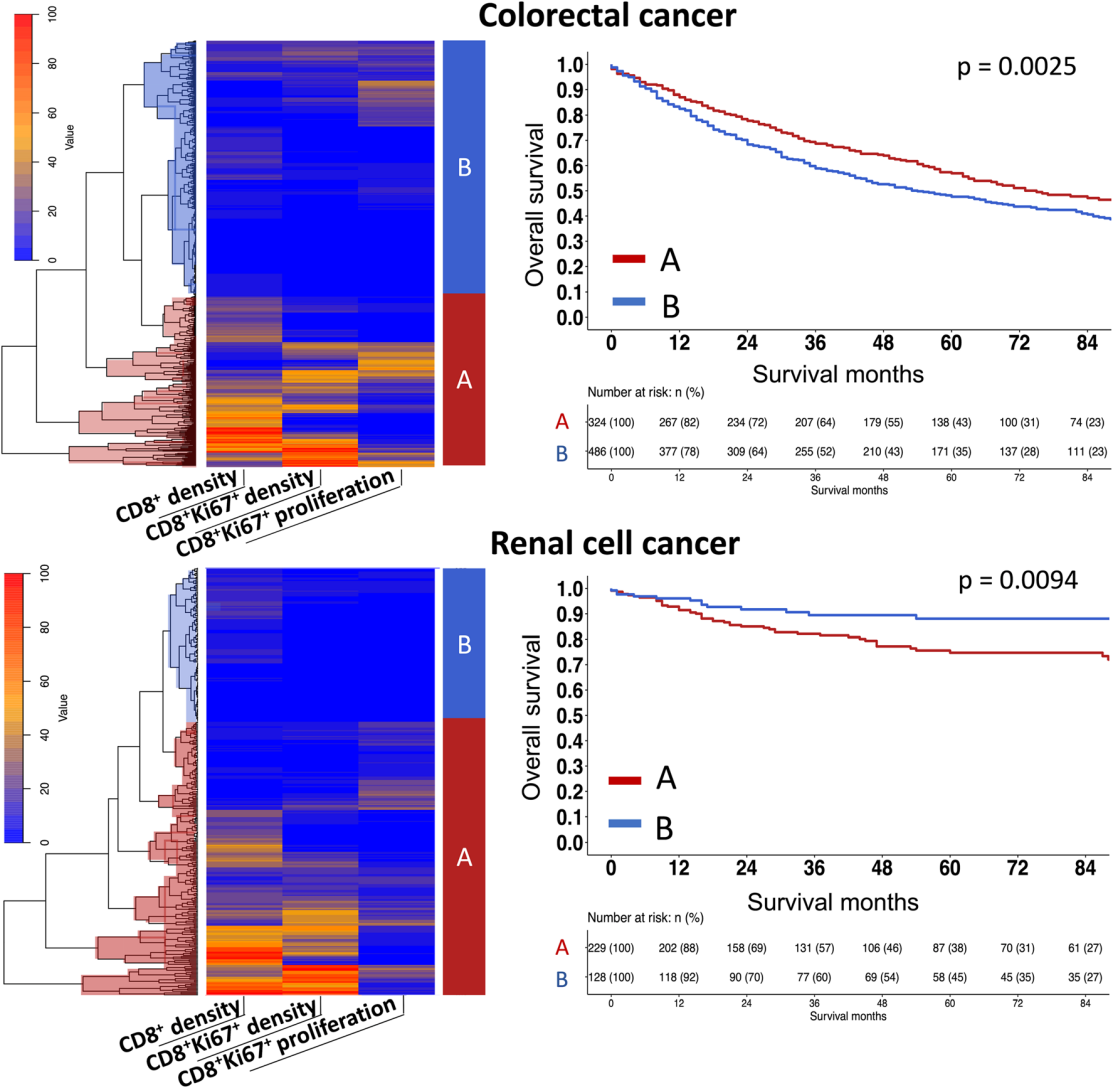
Unsupervised cluster analysis segregates colorectal (top) and renal cell cancer patients (bottom) into the inflamed (**A**) and non-inflamed (**B**) immune phenotypes. Both immune phenotypes show a significant link to overall survival (right)

To search for interpatient differences and similarities in the measured T cell densities and proliferation rates, we applied an unsupervised clustering approach. In colorectal cancer, two major clusters were identified (A: n = 324, B: n = 486). Survival analysis of the clustered patients revealed that cluster A was linked to a particularly good prognosis (*p* = 0.0025, Fig. 3). Cluster A was characterized by elevated CD8^+^/CD8^+^Ki67^+^ T cell densities and represents an inflamed phenotype, while cluster B was characterized by the absence of cytotoxic T cell infiltration (non-inflamed phenotype). Although, two major cluster were also identified in renal cell cancer (A: n = 229, B: n = 128), the inflamed cluster A was found to be associated with unfavourable prognosis and the non-inflamed cluster B with a prolonged overall survival (*p* = 0.0094, Fig. 3).

## Discussion

A standardized assessment of the density and proliferation rate of CD8+Ki67+ cytotoxic T lymphocytes, across six different tumor types, revealed an inverse prognostic impact of these parameters in colorectal and renal cell cancer.

Finding a striking link between elevated CD8^+^ cell densities and favorable tumor parameters in colorectal cancer is in line with earlier studies and can be seen as a validation of our analysis approach. For example, using multiplex fluorescence IHC staining of TMAs Hu et al. showed a significantly longer survival of patients with elevated CD8^+^ densities by manual cell counting in 276 colorectal cancers [27]. In addition, an increased overall and disease-free survival were found in colorectal cancer patients with a high CD8^+^ cell density in all analyzed tumor compartments (i.e., center of the tumor and invasive margin) across several studies analyzing colorectal cancers [2, 28]. Overall, the available data suggest that colorectal cancer is the cancer type with the strongest favorable prognostic impact of CD8^+^ cells. Data also suggest some favorable clinical impact of CD8^+^ cells in other cancer types such as breast [29], gastric [30], ovarian [31], endometrial [32], esophagus [33] and pancreatic [34] cancer. Such findings are in line with the “dogma” of an improved survival of patients showing an inflamed tumor microenvironment [6, 35, 36]. The fact that we found some evidence for a clinical role of CD8^+^ cells only for some cancer types, but not for ovarian, breast and pancreatic cancer, may reflect that TIL quantification is clinically less relevant in these tumors. Moreover, our data revealed an inverse relevance of TILs in RCC. In RCC, a high CD8^+^ density was tightly linked to unfavorable clinicopathological data and poor patient outcome. This fits well with two earlier studies reporting a poor prognosis for RCCs with a high number of TILs in 221 and 135 RCC patients analyzed [16, 17].

To systematically explore the role of proliferating CD8^+^ cytotoxic T cells in the cancer microenvironment, the proliferative activity of CD8^+^ cytotoxic T lymphocytes was measured in 3980 cancer samples from 6 different tumor entities. Overall, the number of proliferating CD8^+^ T cells was significantly associated with the total number of CD8^+^ T cells. This supports the concept that the degree of cytotoxic T cell infiltration in the tumor microenvironment is mainly driven by its local proliferation and less by a secondary infiltration of T cells originating from regional lymph nodes. This is also in line with earlier data showing an increased proliferation rate of cytotoxic T cells in the cancer microenvironment as compared to the “base line” proliferation rate in normal tissues [37]. It is intuitive and has been shown in most cancer types, that an increased proliferative activity of cytotoxic T cells results in high levels of cytotoxic activity and cancer cell killing [38]. Sufficient intratumoral or peritumoral expansion of cytotoxic T cells is likely to be critical for immunologic cancer control, because the success of the Tcell response might dependent on the ratio of active lymphocytes per tumor cells. In one study, the killing capacity of CD8^+^ T cells was shown to be limited to 2–16 cells per day [11]. It is unclear, however, for how long CD8^+^ T cells can assure this killing capacity. Although, it is generally accepted that proliferating CD8^+^ cytotoxic T cells are important for T cell responses [39], it is still unclear to what extent proliferating CD8^+^ T cells exert an anti-tumor effector function. Previous studies analyzing the effector function of proliferating CD8^+^ lymphocytes using flow cytometry [40–43], in vitro models [38], mouse models [44, 45], immunohistochemistry [15, 18, 46] and multiplex fluorescence immunohistochemistry have come to different conclusions [22]. For example, Ganesan et al. analyzed the anti-tumor activity of the cytotoxic T cell subsets in lung cancer and found an enhanced anti-tumor cytotoxicity of proliferating tissue-resident memory CD8^+^ cells [43]. In contrast, Golubovskaya et al. suggested that an increased proliverative activity of CD8^+^ cytotoxic T cells may be linked to a downregulation of effector T cell differentation [47].

The statistical associations with tumor parameters obtained for CD8^+^Ki67^+^ cells were largely comparable to the results on CD8 quantitation alone. This was expected based on the strong correlation of both parameters. Accordingly, a high proliferative activity of CD8^+^ cells was strikingly linked to a favorable outcome in colorectal cancer. Similar data have recently been reported by Imaizumi et al. using multiplex fluorescence IHC on 188 rectal cancers [22]. Mlecnik et al. also found a longer disease-free survival in colorectal cancer patients with an increased T cell proliferation [48]. Again, our analyses revealed an inverse correlation for RCC, where an increased proliferative activity of CD8^+^ cytotoxic Tcells was linked to a poor prognosis. In agreement with our results, a recent study suggested an association between an increased overall and cancer-related mortality and a high fraction of proliferating CD8^+^ cells in a cohort of 78 papillary RCCs [49]. In contrast, Nakano et al. observed an association of a high proliferation rate of CD8^+^ cytotoxic T cells with a longer survival in a cohort of 78 RCCs [16]. The reason for the inverse role of cytotoxic T cells and of proliferating cytotoxic T cells in colorectal cancer and RCC is unclear. It is tempting to speculate, however, that major differences may exist between cancer types in their capability for immune evasion. It may be that an anti-immune mechanism exists in RCC that efficiently protects cancer cells from CD8^+^ cytotoxic T cells, although the immune system reacts by recruiting elevated numbers of lymphocytes that are also capable of intratumoral expansion.

An unsupervised cluster analysis was used to stratify the patients into two groups according to their degree of cytotoxic T cell infiltration and its proliferative activity. The quantification of these parameters in biopsy-like tissue cores measuring 0.6 mm in diameter identified cancers with an inflamed and a non-inflamed immune phenotype. Both immune phenotypes showed a significant and tumor type dependent influence on patient survival. These findings fit well with the conclusions from earlier studies on large sections investigating major cancer immune phenotypes such as “immune-inflamed”, “immune excluded” and “immune desert” (i.e., non-inflamed). Several of these studies have suggested that the immune inflamed and immune excluded phenotypes tended towards a longer survival as compared to the immune desert phenotype [6, 8, 36, 50]. The assumption that immune phenotypes of cancers have a profound impact on their clinical course are in line with the results of an increasing number of publications showing a prognostic relevance of virtually all immune cell subtypes, i.e., CD3^+^ Tlymphocytes [2, 36], CD20^+^ B lymphocytes [51], CD8^+^ cytotoxic T cells, CD8^+^Ki67^+^ cytotoxic T cells, CD4^+^ helper T cells [52], FOXP3^+^ regulatory T cells [53], CD45RO memory T cells [2] and other subsets.

In summary, standardized quantitation of CD8^+^ and CD8^+^Ki67^+^ cytotoxic T cells in six cancer types revealed highly variable relationships with clinicopathological parameters between tumor entities. Colorectal and kidney cancer represent the extremes of the spectrum with a favorable prognosis in colorectal cancers and a poor prognosis in RCCs with high amounts of CD8^+^ and CD8^+^Ki67^+^ cytotoxic T cells. Understanding the interplay between the degree of cytotoxic T cell infiltration and immune evasion mechanisms will be crucial for an improved assessment of cancer immune phenotypes.

## Supplementary Information

ESM 1 **Fig. S1: Digital image analysis workflow.** Area detection (orange), cell segmentation and the visualization of detected CD8^+^ (lightgrey), Ki67^+^ (darkgrey), and Ki67^+^CD8^+^ cells (purple) is shown. 200x magnifications are shown in the insets. **Fig. S2:** Association between CD8^+^ cell density (top), CD8^+^Ki67^+^ cell density (middle), CD8^+^Ki67^+^ proliferation rate (bottom) and progressive free survival (left) and disease specific survival (right) in Renal cell cancer. **Fig. S3:** Association between CD8^+^ cell density (top), CD8^+^Ki67^+^ cell density (middle), CD8^+^Ki67^+^ proliferation rate (bottom) and overall survival in Breast cancer. **Table S1–6:** Patients characteristics. (PDF 3846 kb)

## Data Availability

The datasets used and/or analysed during the current study are available from the corresponding author upon reasonable request.

## Abbreviations

IHC: immunohistochemistry
SD: standard deviation
pN: pathological nodal stage
pT: pathological tumor stage
M*: clinical metastasis stage
G: histologic grade
TILs: tumor infiltrating lymphocytes
TMA: tissue microarray
RCC: renal cell cancer
