# Supplementary material for: Prognostic role of proliferating CD8+ cytotoxic Tcells in human cancers
Source: Cell Oncol (Dordr). 2021 Apr 17;44(4):793–803. doi: 10.1007/s13402-021-00601-4 (PMC8338812; doi:10.1007/s13402-021-00601-4)
Supplement: Supplementary file 1 — Fig. S1: Digital image analysis workflow. Area detection (orange), cell segmentation and the visualization of detected CD8+ (lightgrey), Ki67+ (darkgrey), and Ki67+CD8+ cells (purple) is shown. 200x magnifications are shown in the insets. Fig. S2: Association between CD8+ cell density (top), CD8+Ki67+ cell density (middle), CD8+Ki67+ proliferation rate (bottom) and progressive free survival (left) and disease specific survival (right) in Renal cell cancer. Fig. S3: Association between CD8+ cell density (top), CD8+Ki67+ cell density (middle), CD8+Ki67+ proliferation rate (bottom) and overall survival in Breast cancer. Table S1–6: Patients characteristics. (PDF 3846 kb) [file 13402_2021_601_MOESM1_ESM.pdf]

Cellular Oncology

Prognostic role of proliferating CD8$^+$ cytotoxic T-cells in human cancers

Niclas C. Blessin[1]*, Wenchao Li*[1], Tim Mandelkow[1], Hannah L. Jansen[1], Cheng Yang[1], Jonas B. Raedler[1,2], Ronald Simon[1], Franziska Büscheck[1], David Dum[1], Andreas M. Luebke[1], Andrea Hinsch[1], Katharina Möller[1], Anne Menz[1], Christian Bernreuther[1], Patrick Lebok[1], Till Clauditz[1], Guido Sauter[1], Andreas Marx[3], Ria Uhlig[1], Waldemar Wilczak[1], Sarah Minner[1], Till Krech[1], Christoph Fraune[1], Doris Höflmayer[1], Eike Burandt[1], Stefan Steurer[1]

[1] Institute of Pathology, University Medical Centre Hamburg-Eppendorf, D-20246 Hamburg, Germany

[2] College of Arts and Sciences, Boston University, Massachusetts, USA

[3] Institute of Pathology, Medical Centre Fürth, D-90766 Fürth, Germany

*** These authors have contributed equally to the study**

[a] **Correspondence to:** Ronald Simon, University Medical Centre Hamburg-Eppendorf, Institute of Pathology, Martinistraße 52, D-20246 Hamburg, Germany.

E-mail: r.simon@uke.de

Figure S1: Digital image analysis workflow. Area detection (orange), cell segmentation and the visualization of detected CD8$^+$ (lightgrey), Ki67$^+$ (darkgrey), and Ki67$^+$CD8$^+$ cells (purple) is shown. 200x magnifications are shown in the insets.

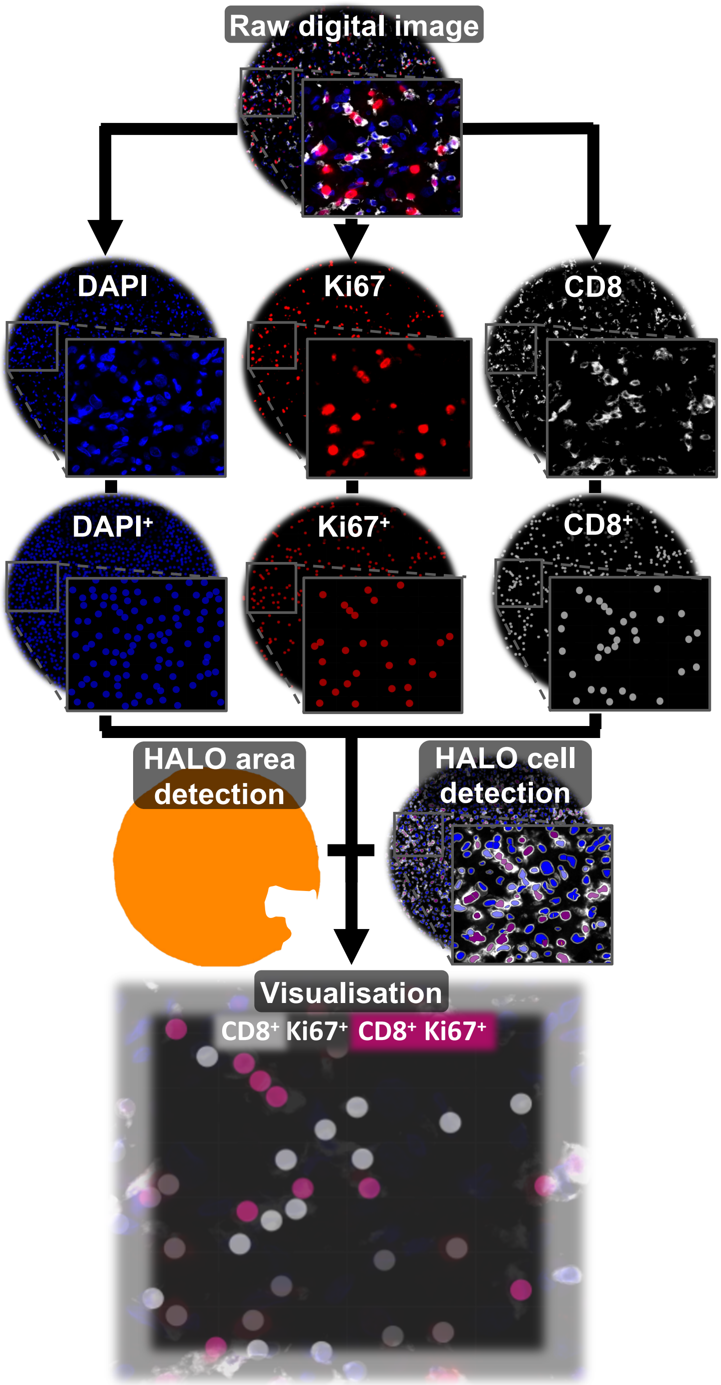

Figure S2: Association between CD8⁺ cell density (top), CD8⁺Ki67⁺ cell density (middle), CD8⁺Ki67⁺ proliferation rate (bottom) and progressive free survival (left) and disease specific survival (right) in Renal cell cancer.

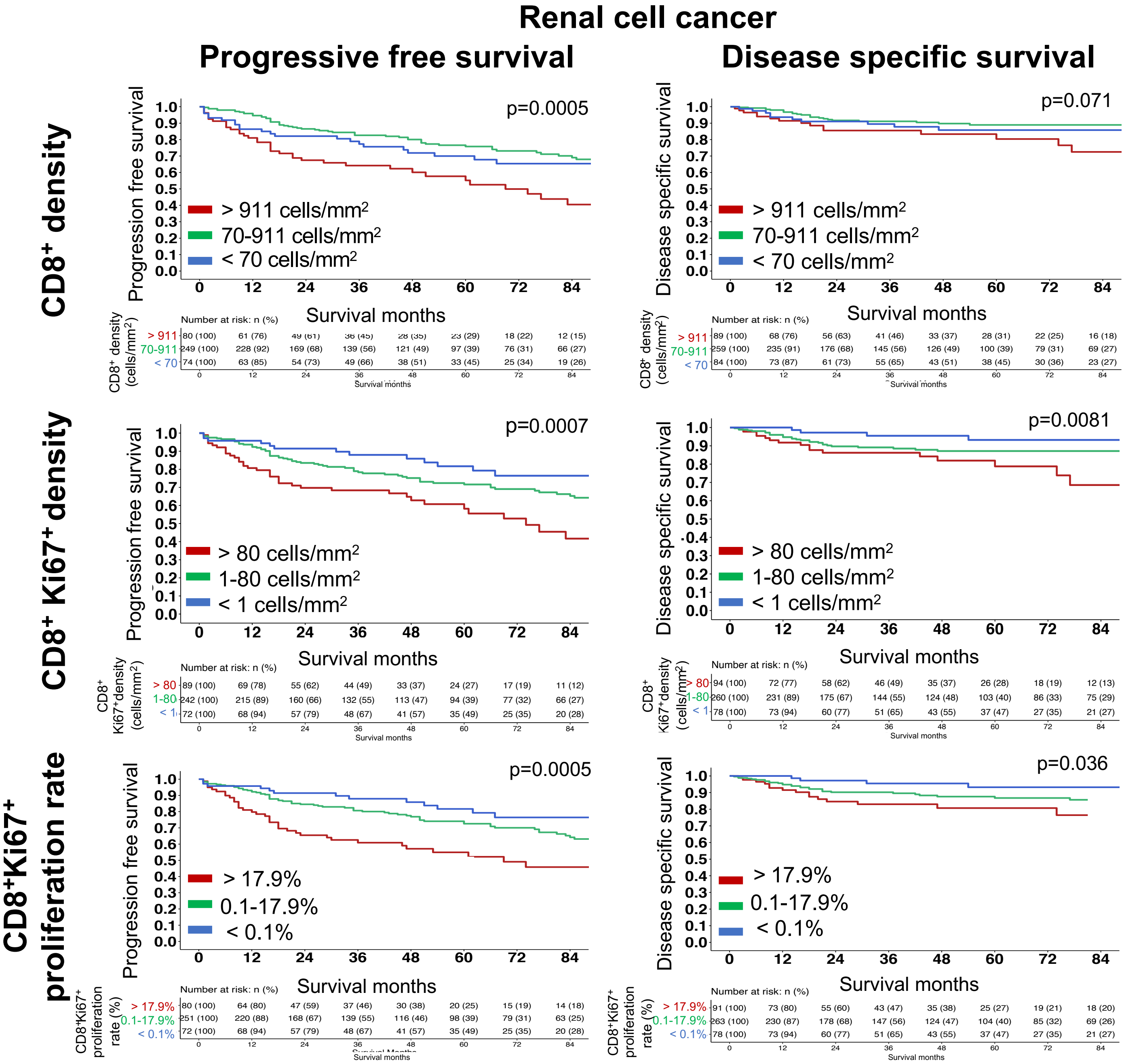

Figure S3: Association between CD8⁺ cell density (top), CD8⁺Ki67⁺ cell density (middle), CD8⁺Ki67⁺ proliferation rate (bottom) and overall survival in Breast cancer.

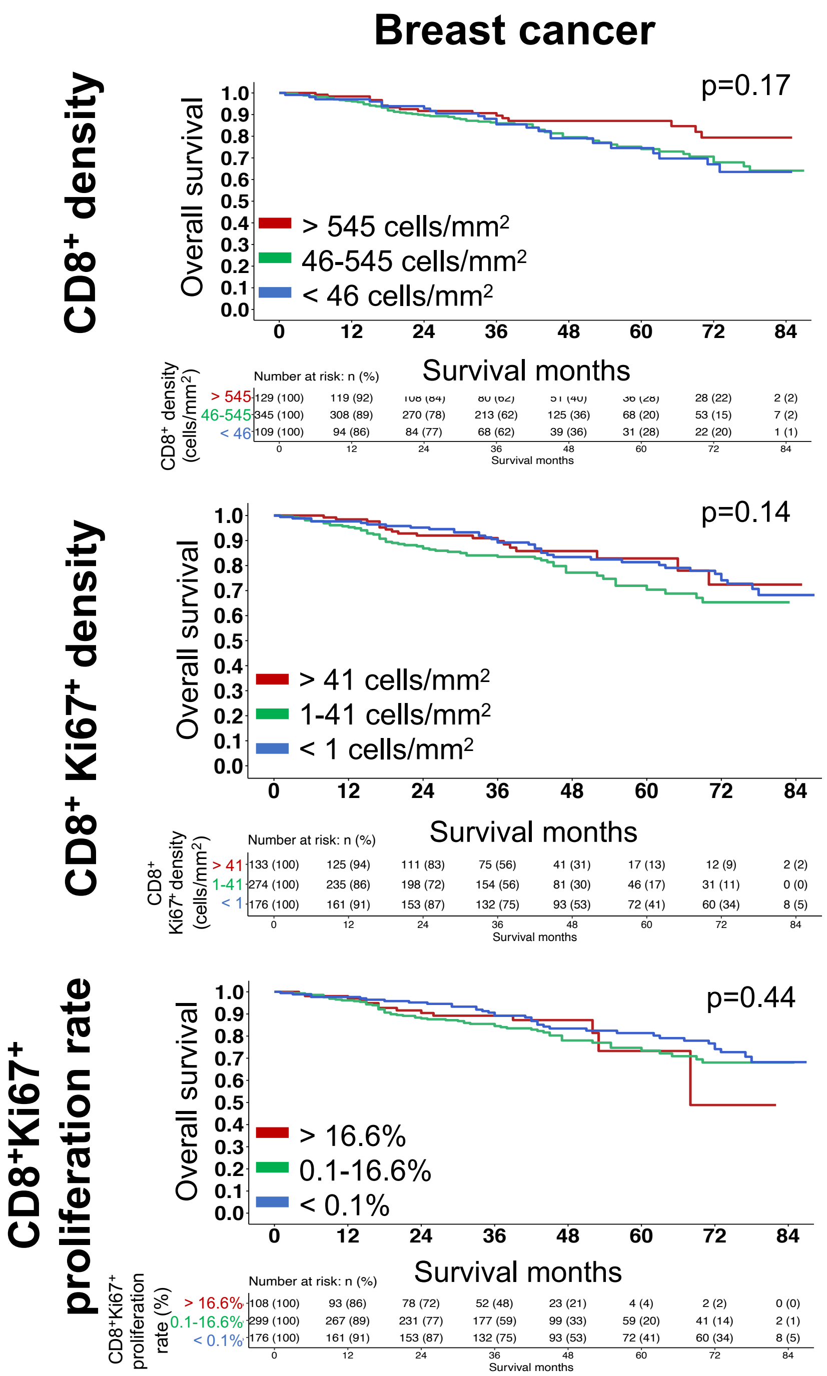

**Supplementary Table 1**: Characteristics of the Colorectal cancer Patients and Relative Risk of Death.		
Patients characteristics	Study cohort on TMA (n=1475)	Overall death among categories
Follow-up - no. (%)	1409 (95.5)	733 (52.0)
Mean/median - months	31.1/23.0	-
Age (median) - years	71	-
pT stage - no. (%)		
pT1	62 (4.2)	11 (17.7)
pT2	203 (13.8)	64 (31.5)
pT3	899 (60.9)	473 (52.6)
pT4	223 (15.1)	169 (75.8)
Missing data	88 (6.0)	-
pN stage - no. (%)		
pN-	711 (48.2)	265 (37.3)
pN+	651 (44.1)	440 (67.6)
Missing data	113 (7.7)	-
Grade - no. (%)		
G1	31 (2.1)	6 (19.4)
G2	1177 (79.8)	610 (51.8)
G3	177 (12.0)	103 (58.2)
Missing data	90 (6.1)	-
Histology type - no. (%)		
Adenocarcinoma	1261 (85.5)	660 (52.3)
Mucinous carcinoma	119 (8.1)	56 (47.1)
Medullary carcinoma	5 (0.3)	4 (80.0)
Signet ring carcinoma	5 (0.3)	4 (80.0)
Missing data	85 (5.8)	-

**Supplementary Table 2:** Characteristics of the Renal cell cancer Patients and Relative Risk of Death.				
Patients characteristics	Study cohort on TMA (n=1809)	Overall death among categories	Tumor specific death among categories	Tumor progression among categories
Follow-up (OS/DSS/PFS) - no. (%)	1174/1174/1077	206 (17.5)	96 (8.2)	268 (24.9)
Mean/median (OS;DSS;PFS) - months	42.7/22.5; 26.1/16.0; 47.8/31.0	-	-	-
Age (median) - years	64	-	-	-
pT stage - no. (%)				
pT1	998 (55.2)	85 (8.5)	21 (2.1)	86 (8.6)
pT2	223 (12.3)	30 (13.5)	17 (7.6)	43 (19.3)
pT3	390 (21.6)	82 (21.0)	53 (13.6)	131 (33.6)
pT4	18 (1.0)	8 (44.4)	4 (22.2)	11 (61.1)
Missing data	180 (10.0)	-	-	-
pN stage - no. (%)				
pN-	232 (12.8)	51 (22.0)	26 (11.2)	75 (32.3)
pN+	59 (3.3)	26 (44.1)	17 (28.8)	44 (74.6)
Missing data	1518 (83.9)	-	-	-
ISUP - no. (%)				
ISUP 1	398 (22.0)	35 (8.7)	10 (2.5)	29 (7.3)
ISUP 2	537 (29.7)	54 (10.1)	19 (3.5)	61 (11.4)
ISUP 3	469 (25.9)	81 (17.3)	45 (9.6)	117 (25.0)
ISUP 4	100 (5.5)	27 (27.0)	18 (18.0)	49 (49.0)
Missing data	305 (16.9)	-	-	-
Fuhrmann - no. (%)				
Fuhrmann 1	72 (4.0)	3 (4.2)	2 (2.8)	6 (8.3)
Fuhrmann 2	851 (47.0)	83 (9.8)	25 (2.9)	81 (9.5)
Fuhrmann 3	480 (26.5)	82 (17.1)	47 (9.8)	118 (24.6)
Fuhrmann 4	110 (7.3)	30 (27.3)	19 (63.3)	53 (48.2)
Missing data	296 (19.6)	-	-	-
Thoenes - no. (%)				
Thoenes 1	497 (27.5)	42 (8.5)	15 (3.1)	37 (7.4)
Thoenes 2	839 (46.4)	103 (12.3)	46 (5.5)	141 (16.8)
Thoenes 3	177 (9.8)	53 (30.0)	32 (18.1)	80 (45.2)
Missing data	296 (16.4)	-	-	-
Histology type - no. (%)				
Clear cell renal cell carcinoma	1176 (65.0)	159 (13.5)	214 (18.2)	72 (6.1)
Papillary renal cell carcinoma	270 (14.9)	29 (10.7)	27 (10.0)	13 (48.1)
Chromophobe renal cell carcinoma	101 (5.6)	3 (3.0)	10 (9.9)	1 (0.9)
Others	285 (14.2)	21 (8.1)	10 (3.9)	23 (8.9)
Mising data	4 (0.3)	-	-	-

**Supplementary Table 3:** Characteristics of the Breast cancer Patients and Relative Risk of Death.		
Patients characteristics	Study cohort on TMA (n=1566)	Overall death among categries
Follow-up - no. (%)	844 (53.9)	175 (20.7)
Mean/median - months	33.1/30.0	-
Age (median) - years	63	-
pT stage - no. (%)		
pT1	778 (49.7)	42 (5.4)
pT2	556 (35.5)	75 (13.5)
pT3	57 (3.6)	17 (29.8)
pT4	79 (5.0)	27 (34.2)
Missing data	96 (6.1)	-
pN stage - no. (%)		
pN-	685 (43.7)	56 (8.2)
pN+	462 (29.5)	84 (18.2)
Missing data	419 (26.8)	-
Grade - no. (%)		
G1	234 (14.9)	13 (5.6)
G2	826 (52.7)	90 (10.9)
G3	467 (29.8)	70 (15.0)
Missing data	39 (2.5)	-
PD-L1 status		
PD-L1 negative	862 (55.0)	106 (12.3)
PD-L1 weak	51 (3.3)	3 (5.9)
PD-L1 moderate	7 (0.4)	0 (0.0)
PD-L1 strong	2 (0.1)	0 (0.0)
Missing data	644 (41.1)	-
Histology type - no. (%)		
NST	1256 (80.2)	134 (10.7)
Lobular carcinoma	171 (10.9)	26 (15.2)
Mucinous carcinoma	36 (2.3)	6 (16.7)
Medullary carcinoma	11 (0.7)	0 (0.0)
Tubular carcinoma	9 (0.6)	2 (22.2)
Basal cell carcinoma	8 (0.5)	3 (37.5)
Papillary carcinoma	4 (0.3)	0 (0.0)
Missing data	71 (4.5)	-

NST Invasive carcinoma of no special type

**Supplementary Table 4:** Characteristics of the Gastric cancer Patients	
Patients characteristics	Study cohort on TMA (n=384)
pT stage - no. (%)	
pT1	29 (7.6)
pT2	45 (11.7)
pT3	163 (42.4)
pT4	147 (38.2)
Missing data	0 (0.0)
pN stage - no. (%)	
pN-	100 (26.0)
pN+	280 (72.9)
Missing data	4 (1.0)
Clinical metastasis stage - no. (%)	
M0*	315 (82.0)
M1*	54 (14.1)
Missing data	15 (3.9)
Histology type - no. (%)	
Intestinal type	92 (24.0)
Diffuse type	90 (23.4)
Mixed type	61 (15.9)
Missing data	141 (36.7)

**Supplementary Table 5:** Characteristics of the Ovarian cancer Patients	
Patients characteristics	Study cohort on TMA (n=607)
pT stage - no. (%)	
pT1	133 (21.9)
pT2	62 (10.2)
pT3	328 (53.9)
Missing data	84 (13.8)
pN stage - no. (%)	
pN-	160 (26.3)
pN+	217 (35.7)
Missing data	230 (37.9)
Histology type - no. (%)	
Serous	427 (70.2)
Mucinous	44 (7.2)
Endometrioid	43 (7.1)
MMMT	30 (4.9)
Clear cell	27 (4.4)
Others	36 (5.9)
Missing data	1 (0.2)

MMMT Malignant mixed mesodermal tumor

**Supplementary Table 6:** Characteristics of the Pancreatic cancer Patients	
Patients characteristics	Study cohort on TMA (n=599)
pT stage - no. (%)	
pT1	18 (3.0)
pT2	92 (15.4)
pT3	430 (71.8)
pT4	43 (7.2)
Missing data	16 (2.7)
pN stage - no. (%)	
pN-	130 (21.7)
pN+	443 (74.0)
Missing data	26 (4.3)
Grade - no. (%)	
G1	19 (3.2)
G2	420 (70.1)
G3	128 (21.4)
Missing data	32 (5.3)
Histology type	
Adenocarcinoma	588 (98.2)
Others	10 (1.7)
Missing data	1 (0.2)
